# Appendicitis

**Published:** 1894-10-20

**Authors:** 


### APPENDICITIS.

The vermiform appendix has been considered by some as a mistake on the part of nature. Be this as it may, it can be quite well dispensed with. The term appendicitis has come to include those conditions formerly known as typhlitis, or peri-typhlitis, and has its origin in the fact that experience has taught us that these inflammatory conditions have their starting point in the appendix. Whether it be due to a fæcal concretion or a foreign body, or to retention within the appendix of its own secretion, due to distortion or adhesions, it is now recognised as a distinct disease. Not only so. From the very nature of this affection it must often first come under the notice of the physician, and whilst it has always been considered essentially a medical disease, it is becoming more and more evident that it ought rather to come under the domain of the surgeon. In a disease at once so variable and so treacherous, there is often no time to be lost. Under rest and careful dieting a mild case may proceed favourably enough. But we may have symptoms of the severest type from the very onset, such as great pain, marked rigidity, and abdominal distension, giving every sign of perforation. In such a case operation may be the patient's only chance. Unfortunately we are not yet in a position to say what cases will recover without operation, judging from the symptoms. Nor are statistics as reliable as in some other affections, for if it be said that half the cases recover without operation, this is only true of the first or second day of the disease. "Only a small proportion of cases that have reached the third or fourth day without decided improvement in the symptoms, recover without operative interference." (Rushmore). That recovery may follow spontaneous rupture into the bowel is quite true. But are we justified in running the risk? Even if recovery does take place we have to remember the danger of recurrence and the possible sudden onset of suppurative inflammation while the patient is travelling or beyond surgical assistance.

The suppurative process, the ulceration and gangrene, the general septic peritonitis so often associated with this disease, at once mark it out as essentially a surgical affection. Indeed it is becoming more and more evident that appendicitis should be considered surgical from the very first in view of the possibility of septic peritonitis which is the greatest danger we have to fear. At first an ulcerative rather than a gangrenous process, the general inflammation in appendicitis may be due to an extension from its original seat, or to an infection through the lymphatics. Even so soon as twenty-four hours after the initial pain it has been found that purulent lymph had already begun to form. What is required, then, is an early and exact diagnosis.

The most reliable symptom seems to be tenderness at the "McBurney point," *i.e.*, two inches from the right anterior iliac spine, on a line from it to the umbilicus. The indications of the thermometer appear to be of little value, but the sudden onset, the tension of the muscles, the ill-defined tumour in the right iliac region, the constipation, and lastly the facial expression, are all points of the greatest importance. In a mild case liquid diet, evacuation of the bowels by enemata, the local application of cold, warmth, or leeches will be found efficacious, and, above all, rest in bed is absolutely essential in order to prevent recurrence. But in a severer type of the disease something more may be required than mere expectant treatment. If the acute symptoms do not subside within twenty-four or thirty-six hours operation offers the best prospect of recovery, and, when the symptoms are extremely urgent, the same line of treatment will, even at an earlier stage, prove less dangerous then delay. Our duty then, as practitioners, is to endeavour to make an exact diagnosis as early as possible, and, if no improvement takes place during the first two days of the disease, to remember the importance of allowing the surgeon to see the case at once, in order that he may be in a position to judge whether operative interference be necessary or not; for although operation is not always called for, it is in many cases the only remedy.

